# The Effect of Intravenous Administration of Alprostadil on Circulating Microvesicles in Patients with Chronic Limb-Threatening Ischemia (CLTI): A Preliminary Study

**DOI:** 10.3390/jcm15135132

**Published:** 2026-07-01

**Authors:** Cosmin Alexandru Buzilă, Miruna Nemecz, Ionel Droc, Liviu Stan, Constantin Ghiaţău, Alin Constantin Anăstăsoaie, Alice Elena Munteanu, Adriana Georgescu

**Affiliations:** 1 Cardiovascular Surgery Department, ’Dr. Carol Davila’ Central Military Emergency University Hospital, 010825 Bucharest, Romania; 2 Department of Pathophysiology and Cellular Pharmacology, Institute of Cellular Biology and Pathology ‘Nicolae Simionescu’ of Romanian Academy, 050568 Bucharest, Romania; 3 Department of Medical-Surgical and Prophylactical Disciplines, Faculty of Medicine, ‘Titu Maiorescu’ University, 031593 Bucharest, Romania, 040051 Bucharest, Romania

**Keywords:** alprostadil, microvesicles, biomarkers, chronic limb threatening ischemia, peripheral artery disease, amputation free survival

## Abstract

**Background/Objectives**: The treatment of patients with advanced peripheral arterial disease, such as chronic limb-threatening ischemia (CLTI), often requires more than the use of conventional vasodilators and revascularization procedures, when these are feasible. Alprostadil is typically administered intravenously to treat these patients. However, existing studies reported conflicting results regarding the efficacy of the aforementioned treatment. It is hypothesized that, based on clinical experience, this therapy may exhibit a benefit for patients with CLTI, promoting ulcer healing, alleviating pain, and improving limb salvage rates. Consequently, the concept of employing blood-circulating microvesicles (MVs) as biomarkers for evaluating treatment effectiveness and estimating clinical prognoses has been proposed. **Methods**: These were analyzed in a group of patients with CLTI before and during alprostadil treatment, with and without revascularization procedures, to assess their correlation with clinical outcomes. **Results:** Our data indicated that CLTI results in an increase in circulating levels of MVs, as well as in specific subtypes of plasma MVs—MVs derived from endothelial cells (EMVs), platelets (PMVs), leukocytes (LeMVs), monocytes (MMVs), and erythrocytes (ErMVs)—compared to levels observed in the plasma of healthy individuals. Furthermore, treatment with alprostadil was associated with a significant reduction in plasma levels of total MVs, as well as EMVs, PMVs, LeMVs, and MMVs. In contrast, revascularization procedures do not appear to result in a reduction in circulating MV levels. These results are consistent with the clinical improvements observed in CLTI patients treated with alprostadil. **Conclusions**: In summary, the findings of this preliminary study suggest that blood-circulating MVs, including specific subtypes such as EMVs, PMVs, LeMVs, and MMVs, may have potential as biomarkers for assessing the response to alprostadil treatment in patients with CLTI.

## 1. Introduction

The term “peripheral artery disease (PAD)” refers to the structural and functional impairment of arteries that supply tissues other than those of the heart and brain [1]. Approximately 200 million people worldwide suffer from PAD, with age being one of the primary risk factors for the disease’s prevalence. Consequently, the prevalence of PAD increases by 10–25% in individuals older than 55 years and by up to 40% in those older than 80 years. PAD has been demonstrated to have a significant impact on patients’ quality of life, often resulting in chronic limb-threatening ischemia (CLTI), the necessity of limb amputation, and, in some cases, even mortality.

Even in patients where open or endovascular revascularization is a viable treatment option, PAD can result in the loss of tissue or limbs. More, when no other viable treatment options are feasible, also known as non-revascularizable patients, the best medical therapy is the sole hope of avoiding amputation.

Prostanoids are prescribed for patients with advanced PAD to address symptoms stemming from ischemia and to promote the healing of trophic lesions prior to or following revascularization procedures. These molecules have been demonstrated to exhibit vasodilator effects, decrease inflammation, and help prevent thrombosis. The existing literature on the subject remains contentious, with conflicting findings regarding the efficacy of prostanoids in the therapeutic management of patients with PAD. Although prostanoids have been associated with modest improvements in ischemic rest pain and ulcer healing among patients with CLTI who are not eligible for revascularization, current evidence from the Cochrane Database of Systematic Reviews indicates no significant reduction in amputation risk or mortality, and their use is frequently limited by treatment-related adverse effects [2].

The involvement of extracellular vesicles (EVs) in the pathogenesis of atherosclerotic diseases (such as coronary artery disease and cerebrovascular disease) has been previously described. EVs are heterogeneous membrane-enclosed structures secreted by a wide range of cell types, facilitating intercellular communication through the delivery of biologically active cargo to recipient cells. According to their size, EVs can be categorized as large EVs, including microparticles (MPs) or microvesicles (MVs) (100–1000 nm), and small EVs, commonly termed exosomes (40–100 nm). The release of EVs by activated or apoptotic cells has been observed in both tissues and body fluids. These vesicles have been demonstrated to exert a detrimental influence on intercellular communication [3,4]. The composition of their cargo is contingent upon the particular pathophysiological condition that initiated their release during the packaging and secretion process.

Recent studies have highlighted the crucial role of exosomes derived from various cell types in the development and progression of atherosclerosis through the modulation of cholesterol transport, inflammation, apoptosis, and other metabolic processes in recipient cells [5]. Nevertheless, the specific role of EVs in PAD remains incompletely understood and warrants further investigation [6]. Notably, EVs exhibit diverse biological functions, including proangiogenic activity, as well as both proatherogenic and antiatherogenic effects, underscoring their complex involvement in vascular pathophysiology.

Among the different subtypes of EVs, microvesicles (MVs) have attracted considerable interest owing to their distinctive molecular composition and biological functions. A hallmark feature of MVs is the externalization of phosphatidylserine (PS), a process triggered by increased intracellular calcium levels and commonly associated with cellular activation or apoptosis [7]. Formed through outward budding and fission of the plasma membrane, MVs retain membrane receptors and cytosolic components from their parent cells, thereby reflecting the phenotypic characteristics of their cellular origin [8]. Accordingly, endothelial cell-derived microvesicles (EMVs), platelet-derived microvesicles (PMVs), and leukocyte-derived microvesicles (LMVs) express specific surface markers, including CD144, CD41, and CD18, respectively [9,10]. In addition, MVs carry a broad range of bioactive cargo, such as proteins, DNA, RNA, and microRNAs (miRNAs), enabling them to modulate intercellular signaling and vascular homeostasis [11]. In the context of PAD, circulating MVs have been proposed as markers of platelet activation and have also been implicated in angiogenic and inflammatory pathways, particularly those derived from endothelial cells and leukocytes [12,13,14,15,16,17].

Given the emerging evidence implicating MVs in the pathophysiology of PAD and their potential role as biomarkers of vascular dysfunction, inflammation, and angiogenesis, further investigation into their clinical significance is warranted. Therefore, the objective of this preliminary study was to assess the impact of alprostadil administration on circulating plasma MV levels in patients diagnosed with CLTI. Furthermore, we explored whether circulating MV levels could serve as potential predictors of treatment response to alprostadil in this patient population.

## 2. Materials and Methods

### 2.1. Patients and Control Subjects

The present preliminary study was conducted with the approval of the Ethics Committee of the Central Military Emergency University Hospital “Dr. Carol Davila” in Bucharest, Romania (approval registration number 427/2020). Ten (10) patients diagnosed with chronic limb-threatening ischemia (CLTI) from the Department of Vascular Surgery at the Central Military Emergency University Clinical Hospital “Dr. Carol Davila” in Bucharest, Romania, were included in the study. Additionally, ten non-CLTI subjects (healthy individuals, C group) were included in the study to quantify a basal level of MVs and to serve as a control group for the CLTI patients.

In order to be included in the study and subsequently undergo evaluation, written informed consent was obtained from each patient. All procedures were conducted in accordance with the Declaration of Helsinki (1975), with subsequent revisions made in 2008. Participants were randomly allocated to the study groups. The inclusion criteria for the study were as follows: patients over 18 years of age, both diabetic and non-diabetic, diagnosed with chronic limb threatening ischemia of the upper or lower limb (stages III-IV Fontaine; Rutherford).

The exclusion criteria encompassed the contraindications enumerated by the manufacturer of the pharmaceutical agent utilized in the study: alprostadil (prostaglandin E1)/Pridax (GebroPharma GmbH, Fieberbrunn, Austria). The exclusion criteria included advanced heart disease (ischemic coronary disease and/or moderate or moderate–severe valvular disease (class III or IV NYHA), hypotension, or known allergies to drug components.

Data were obtained from the observation sheets, respectively, as the case may be, from the patient’s personal archive. The evaluation protocol included a vascular surgery exam and paraclinical investigations, as well as a cardiac exam that included transthoracic echocardiography and electrocardiogram. The determination of the alprostadil dosage was based on several factors: patient fragility, the presence of comorbidities, the severity of trophic lesions, and clinical manifestations. These factors were taken into account to ensure the appropriate therapeutic response, especially when determining the number of treatments.

To assess treatment efficiency and clearly correlate MV levels, we divided the CLTI patients as follows: (1) Patients with critical limb ischemia (CLTI) receiving only alprostadil (Pridax) (*n* = 5); (2) Patients with CLTI receiving both revascularization and alprostadil (*n* = 5). The study on CLTI patients treated with alprostadil, who underwent or did not undergo revascularization procedures, was conducted at three distinct moments during the treatment administration process. The initial moment was designated as T0, corresponding to the initiation of alprostadil administration (0 weeks). This was used to establish a negative control group (patients with CLTI who did not receive alprostadil or undergo revascularization) which we should refer to determine the effectiveness of the alprostadil treatment. The second moment was defined as T1 (2 weeks), which occurred after two weeks of treatment administration. The third moment was defined as T2 (4 weeks), which occurred after the last dose of alprostadil administration and at the conclusion of the treatment.

The patients were then randomly assigned in a 1:1 ratio to receive surgical or endovascular treatment. All the patients were expected to receive their assigned treatment within 30 days after randomization. An investigator with expertise in surgical bypass procedures had to agree with another investigator with expertise in endovascular revascularization procedures that clinical equipoise existed in the randomization of each patient.

### 2.2. Clinical Evaluation

For each patient, a tolerance test was performed according to internal procedures. On the first day of treatment, an ampoule of alprostadil (20 micrograms of alprostadil per vial) (Pridax, GebroPharma GmbH, Austria), diluted in 0.5 L of normal saline solution (B. Braun, Melsungen AG, Germany), was administered, with an infusion time of approximately 2 h. During the procedure, vital signs, including heart rate, oxygen saturation, blood pressure, and electrocardiogram, were closely monitored. If the tolerance test was successful, the following day, two ampoules of alprostadil were administered, diluted in 0.5 L of saline, and infused with the same dose. Depending on the therapeutic response and the patient’s clinical condition, the dose was increased to 3 or 4 ampoules per day, administered in two separate doses, 12 h apart (either two infusions of 2 ampoules each, or an infusion of 2, plus infusion with 1 ampoule, in 0.5 L physiological serum). The treatment was administered for a period of 30 days (30-day cure, 4-week cure). During this period, all patients received the same cumulative dose of alprostadil, and no other type of vasodilator was administered.

### 2.3. Imagistic Investigation

The presence and distribution of arterial lesions was evaluated by Doppler ultrasound and digital subtraction angiography. The following parameters were evaluated: blood pressure, heart rate, complete blood count, claudication index (AC), ankle-brachial index (ABI), rest pain, trophic lesions, survival without amputation. Pain at rest was quantified using a scale of 0 to 10 (0 = no pain at rest, 10 = severe pain at rest unresponsive to analgesic therapy). The ankle-brachial index (ABI) was measured according to the protocols in the literature. The ankle-brachial index is recommended as a first-line hemodynamic test in the non-invasive evaluation of patients with CLTI. The initial T0 value for the ABI assessment was considered as follows: the moment of inclusion in the study (first assessment) for negative control patients (non-vascularized, without treatment with alprostadil) and immediately before the initiation of treatment with alprostadil for patients who followed treatment with alprostadil, with or without revascularization.

### 2.4. Therapeutic Strategy

The presence of trophic lesions was documented, and serial photographs were taken. In accordance with data confidentiality protocols, each patient was assigned a code from P1 to P10. Of the 10 CLTI patients treated with alprostadil, five underwent revascularization procedures and five were treated exclusively with alprostadil. The revascularization procedures (open or endovascular) were performed concomitantly with the initiation of alprostadil treatment.

For patients with trophic lesions and signs of superinfection, the presence and evolution of hematological parameters, such as leukocytosis, were monitored.

The wound management protocol was applied uniformly to all patients. The wounds were cleaned and disinfected with microdacyn (Sonoma Pharmaceuticals, Roermond, The Netherlands) and povidone-iodine (Egis Pharmaceuticals, Roermond, Hungary). Hyaluronic acid and collagenase cream/ointment, or hyaluronic acid with silver sulfadiazine (Fidia Farmaceutici, Bologna, Italy), were used when necessary.

### 2.5. Microvesicle Isolation

Serial centrifugations of peripheral blood collected by venipuncture (on vacutainer tubes with EDTA K3 anticoagulant) were used to isolate circulating MVs from each patient/control subject taken into the study [7]. The first centrifugation was performed at 2500× *g* for 10 min at 4 °C to obtain the platelet-poor plasma (PPP). The next step was to centrifuge 200 µL of PPP at 16,000× *g* for 5 min at 4 °C. This process was used to remove any residual platelets and apoptotic bodies. Therefore, the platelet-free plasma (PFP) was collected in the upper layer of the sample. Finally, the MVs were removed after PFP centrifugation at 20,000× *g* for 90 min at 4 °C. The pellet containing MVs was washed twice (20,000× *g*, 90 min, 4 °C) with phosphate-buffered saline (PBS), re-suspended in PBS (~100 μL), and stored at −80 °C until further analysis [18].

### 2.6. Microvesicle Quantification and Analysis

Microvesicles (MVs) were measured by flow cytometry (Gallios Flow Cytometer, Beckman Coulter Life Sciences, Brea, CA, USA) using stabilized pre-analytical and analytical procedures.

To quantify MVs in circulation, 10 µL of resuspended MVs were mixed with 10 µL of counting beads (1000 beads/µL, 10 µm diameter). Furthermore, to assess the purity of MVs, 10 µL of the sample was incubated with 2.5 µL of the Annexin V-FITC antibody in a solution of 2 mM CaCl_2_. Following a 40-min incubation at room temperature in the dark, the samples were diluted with 100 µL of phosphate-buffered saline (PBS) and analyzed using a flow cytometer for 60 s.

The number of MVs (0.1–1 µm diameter) was calculated using the following formula: MVs as events/µL = [(MV count/bead count) × bead concentration/µL] × MV purity/100, where MV count and bead count were collected from the dot-plot representations (X = forward-scatter intensity, Y = side-scatter intensity) [18,19].

To analyze the levels of specific cell-derived MVs, 10 µL of resuspended MVs was incubated with 2.5 µL of Annexin V-FITC antibody and specific surface marker fluorescent antibodies for 40 min at room temperature in the dark.

The following fluorescent antibodies, all from Santa Cruz Biotechnology, Inc. (Dallas, Texas, CA, USA) were utilized: CD41 (integrin alpha-IIb) serves as a specific marker for platelets; CD144 (VE-cadherin) functions as a specific marker for endothelial cells; CD14 acts as a specific marker for monocytes; CD18 (integrin beta chain-2) serves as a specific marker for leucocytes; and CD235a (glycophorin A) functions as a specific marker for erythrocytes.

Prior to flow cytometric analysis (with a 5000-event cutoff), 100 µL of PBS was added to each sample.

The concentrations of endothelial cell-derived MVs (EMVs: CD144+/AnnexinV+), platelet-derived MVs (PMVs: CD41+/AnnexinV+), leukocyte-derived MVs (LeMVs: CD18+/AnnexinV+), monocyte-derived MVs (MMVs: CD14+/AnnexinV+) and erythrocyte-derived MVs (ErMVs: CD235a+/AnnexinV+) were calculated, and their percentages for double labeling were reported as a percentage of the total plasma MVs (AnnexinV+). The concentration of the total plasma MVs was previously calculated according to the formula presented above.

The flow cytometer was calibrated and size discrimination was performed using standardized size-calibration beads with a diameter of 10 µm to define the submicron particle region. MVs were identified based on their forward scatter (FSC) and side scatter (SSC) characteristics, as well as their fluorescent labeling. When applicable, fluorescence compensation and appropriate controls were used to ensure accurate signal discrimination.

The obtained data were analyzed using Kaluza Flow Cytometry Analysis Software v2.1 (Beckman Coulter Life Sciences, Indianapolis, IN, USA) and expressed as a percentage of MVs double labeled with Annexin V and antibody specific to the cell of origin.

### 2.7. Statistical Analysis

Experiments were performed in triplicate, and the results were expressed as the means ± standard error of the mean (SEM). The differences between the experimental groups were determined using a one-way ANOVA followed by Tukey’s multiple comparison tests. Statistical significance was set at *p* < 0.05 (*, #), *p* < 0.01 (**, ##), *p* < 0.001 (***, ###), using GraphPad Prism 8.0.1.244 software (GraphPad Software Inc., San Diego, CA, USA).

## 3. Results

### 3.1. Demographic and Clinical Characteristics of Patients

The present preliminary study encompasses a total of 10 patients (7 men and 3 women) diagnosed with chronic limb-threatening ischemia (CLTI), and referred to the Department of Cardiovascular Surgery at the Central Military Emergency University Clinical Hospital “Dr. Carol Davila” in Bucharest, Romania. The mean age of the patients at the time of enrollment in the study was 64 years, with a range of 49 to 79 years. The initial visit of the first patient occurred in November 2020, while the final visit of the last patient was recorded in January 2022. Patients were the subject of clinical, biological, and sonographic evaluations at intervals ranging from four days to one year, contingent upon therapeutic compliance, symptomatology, and evolution.

Clinical examinations indicated that treatment with alprostadil (Pridax) is safe for carefully selected patients, with no deaths reported. These preliminary findings are consistent with data reported in the literature [20].

It was also observed that administering alprostadil therapy to patients with CLTI contributes to ulcer healing, pain reduction, and improved limb salvage rates. Patients, regardless of whether they underwent revascularization procedures, were evaluated at three distinct time points during treatment: T0 (week 0, before therapy initiation), T1 (week 2, after two weeks of treatment), and T2 (week 4, after four weeks of treatment).

**Figure 1:**
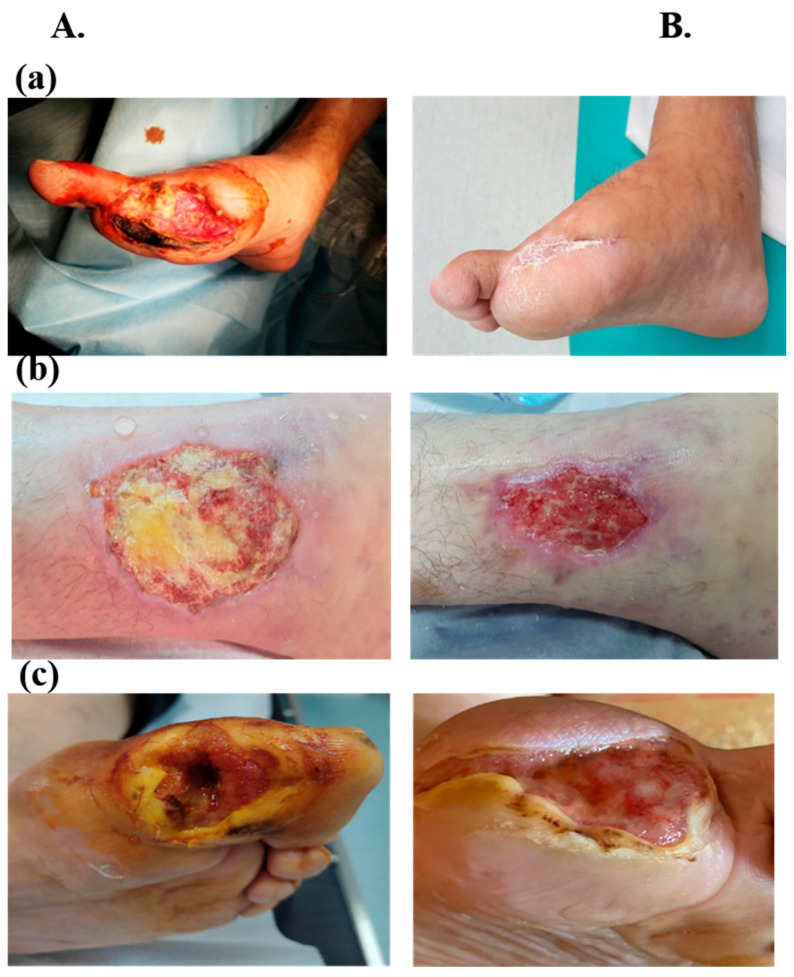
The change in wound status at two time points during alprostadil treatment: (**A**) T0 (week 0, before therapy initiation) and (**B**) T2 (week 4, after four weeks of treatment) for patients with chronic limb-threatening ischemia (CLTI) treated with alprostadil and bypass surgery (above-knee vein graft) (**a**), with alprostadil and endovascular revascularization (transluminal angioplasty and stent implantation in the common iliac artery) (**b**), and without alprostadil administration or revascularization (**c**). These images were obtained at the Central University Military Hospital “Carol Davila”.

A clinical evaluation of the study population revealed that patients treated with alprostadil exhibited superior outcomes (see Figure 1), as evidenced by lower MV levels in comparison with the negative control group (patients with CLTI who did not receive alprostadil or undergo revascularization).

The progression of wound status at two distinct time points during alprostadil treatment is shown in Figure 1A (T0, week 0, before therapy initiation) and Figure 1B (T2, week 4, after four weeks of treatment). The data represent clinical outcomes for patients with CLTI treated with alprostadil (a), with alprostadil and endovascular revascularization (b), or without alprostadil administration and revascularization (c).

**Figure 2:**
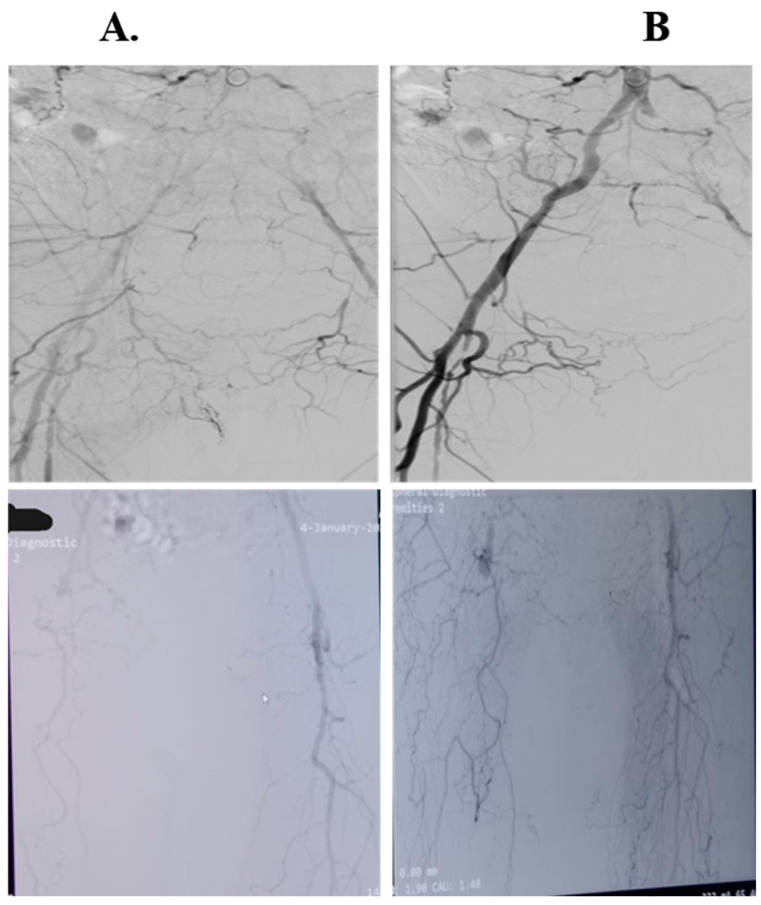
Digital subtraction angiography used to evaluate chronic limb-threatening ischemia (CLTI) patients treated with alprostadil at two distinct time points: T0 (week 0, before therapy initiation) (**A**), and T2 (week 4, after four weeks of treatment) (**B**). These images were obtained at the Central University Military Hospital “Carol Davila”.

As illustrated in Figure 2, digital subtraction angiography was employed to evaluate patients with CLTI who were treated with alprostadil at two distinct time points: T0 corresponds to the pre-therapy initiation phase (week 0) (Figure 2A), while T2 corresponds to the four-week post-therapy initiation phase (week 4) (Figure 2B). The administration of alprostadil resulted in a substantial enhancement in collateral circulation in both lower limbs, extending from the thigh, knee, and calf regions.

A comprehensive documentation of the clinical characteristics and medical histories of patients with chronic limb-threatening ischemia (CLTI) who were included in the study can be found in Table 1.

As shown in Table 1, two patients underwent amputation after alprostadil treatment ended: one major amputation of the calf (four months after T2) and one transmetatarsal amputation (three months after T2). The rest of the patients remained amputation-free at the one-year follow-up. Also, we documented the presence or absence of pain at the end of treatment. Eight patients were pain-free, while the other two had a pain level of 5 out of 10 (1–10), which was lower than at T0 (7 out of 10). Regarding wound closure and epithelialization, the evaluation was qualitative and combined with the quality of life assessment. This information was included in the revised manuscript on page 8.

A list of the key plasma parameters that are monitored in patients diagnosed with CLTl at the T0 time point is provided in Table 2. The T0 time point is defined as -0 weeks prior to the administration of treatment with alprostadil (Pridax).

### 3.2. Quantification and Analysis of Plasma Microvesicles

**Figure 3:**
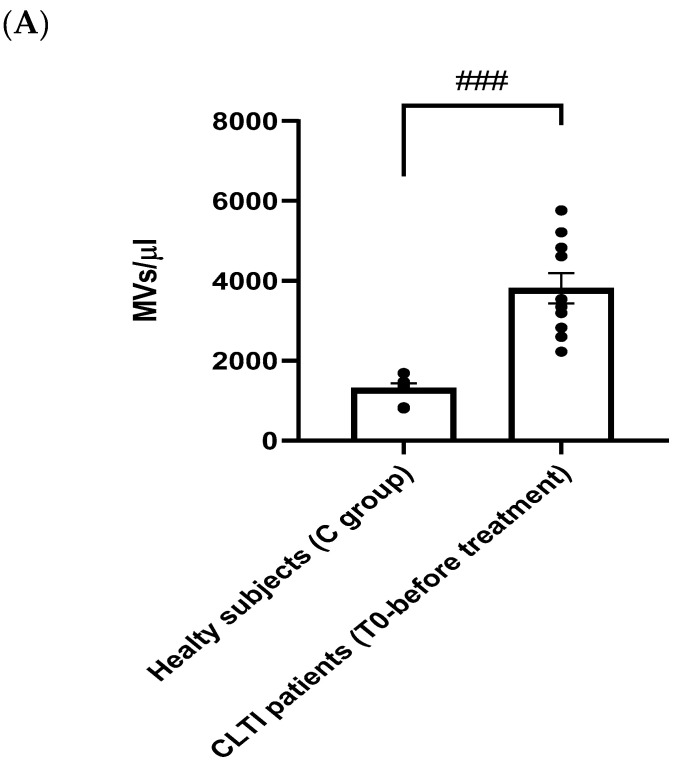

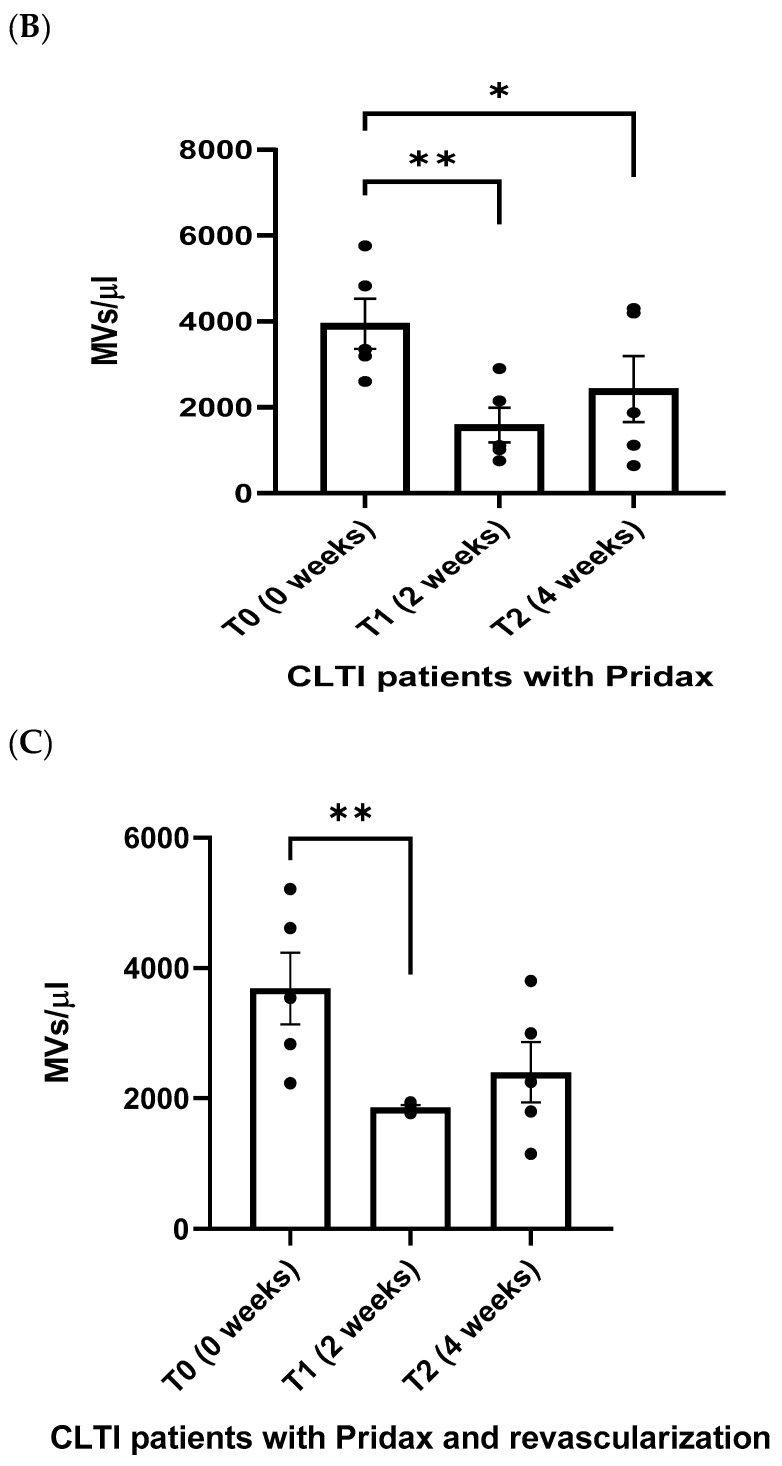
Annexin-V-positive plasma MV concentrations in: (**A**) patients with chronic limb-threatening ischemia (CLTI) and healthy subjects; (**B**) CLTI patients treated with alprostadil (Pridax); (**C**) CLTI patients treated with alprostadil (Pridax) and undergoing revascularization procedure. Data are represented as means ± SEM. * *p* < 0.05 (vs. T0, patients from the Pridax ± revascularization group); ** *p* < 0.01 (vs. T0, patients from the Pridax ± revascularization group); ### *p* < 0.001 (vs. C group) (one-way ANOVA with Tukey’s multiple comparison test).

The levels of plasma MVs in 10 CLTI patients and 10 healthy subjects (C group) were analyzed by flow cytometry (Figure 3A). Plasma samples from the patients with CLTI and healthy individuals were utilized to isolate circulating MVs through a series of centrifugations performed at 4 °C. The analysis of MVs was accomplished by means of flow cytometry. The quantification of purified MVs isolated from both patient and healthy subject plasma was performed using beads of known size and concentration (1000 beads/µL, 10 µm diameter) and an antibody AnnexinV-FITC for a specific marker for MVs: phosphatidylserine (PS). A comparative analysis between these two groups was conducted, and the results are presented in Figure 3A.

Additionally, plasma MVs in 10 CLTI patients treated with alprostadil (Pridax), who underwent or did not undergo revascularization procedures, were assessed at three distinct moments during treatment: T0 (week 0, prior to treatment administration); T1 (week 2, after two weeks of treatment administration); and T2 (week 4, after four weeks of treatment administration) (Figure 3B,C). The initial moment was designated as T0, which corresponded to the start of alprostadil (Pridax) administration (week 0). This was utilized to establish a negative control for the treatment effect.

As illustrated in Figure 3A, prior to the initiation of treatment with alprostadil (T0—0 weeks) in patients with CLTI, the levels of circulating MVs were found to be significantly higher in comparison to those observed in healthy subjects (C group) (Figure 3A).

In the course of a 2-week treatment period (T1) and a 4-week treatment period with alprostadil (T2), patients with CLTI demonstrated a significant reduction in the levels of MVs present in their circulation, in comparison with the levels observed prior to the initiation of treatment (T0) (Figure 3B,C). Furthermore, the levels of plasma MVs in patients with CLTI who received treatment for only two weeks were comparable to the levels of MVs in the plasma of healthy subjects (Figure 3).

*It is important to note that we observed no difference in plasma levels of MVs between CLTI patients treated with alprostadil (Pridax) who underwent revascularization and those treated only with alprostadil. Revascularization intervention does not appear to reduce circulating MV levels* (Figure 3B,C). *Based on these results, subsequent experiments included only CLTI patients treated with alprostadil who did not undergo revascularization.*

*This finding suggests that CLTI may be associated with increased levels of blood-circulating MVs compared to those observed in the plasma of healthy individuals. Furthermore, alprostadil treatment appeared to be associated with a reduction in plasma MV levels, even within the initial two weeks of treatment.*

### 3.3. Quantification and Analysis of Certain Types of Plasma Microvesicles

To analyze MVs according to the types of cells from which they originate in the circulation, the flow cytometry was used.

**Figure 4:**
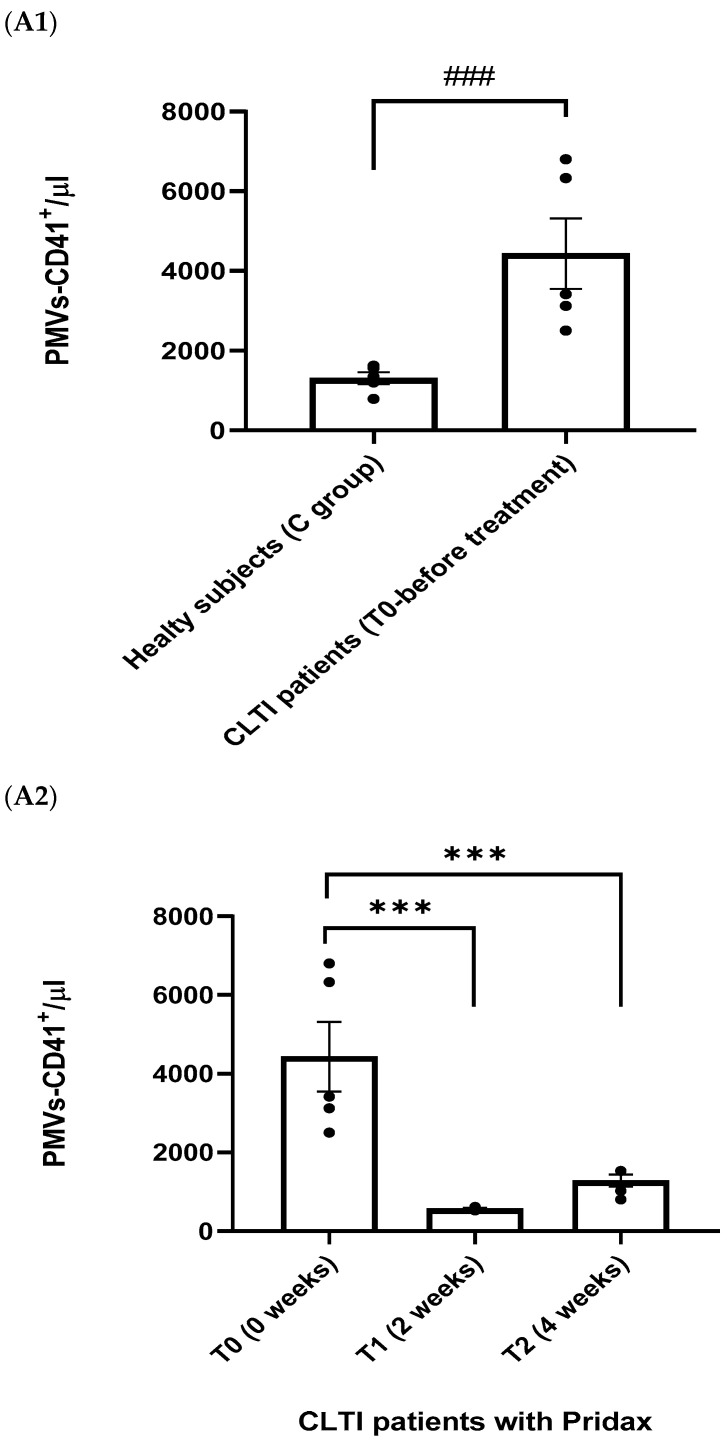

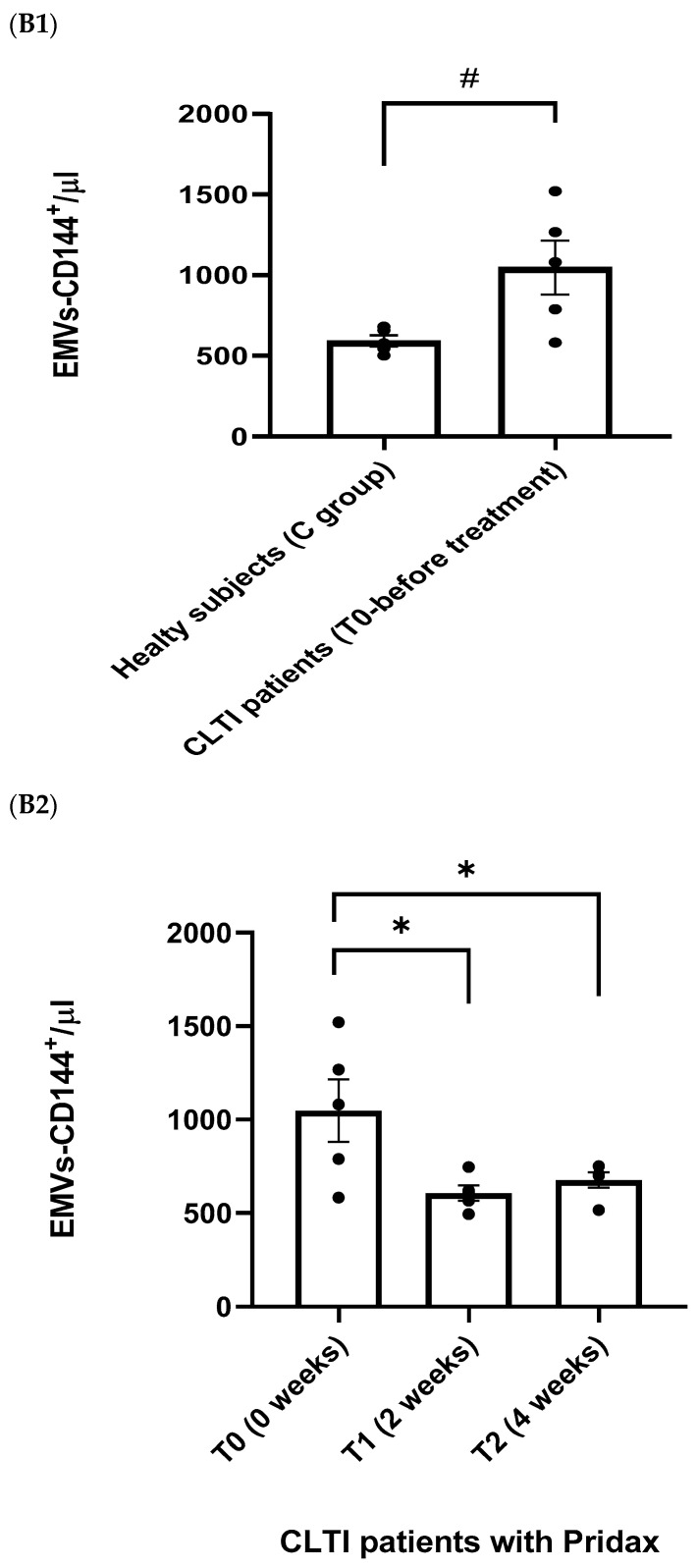

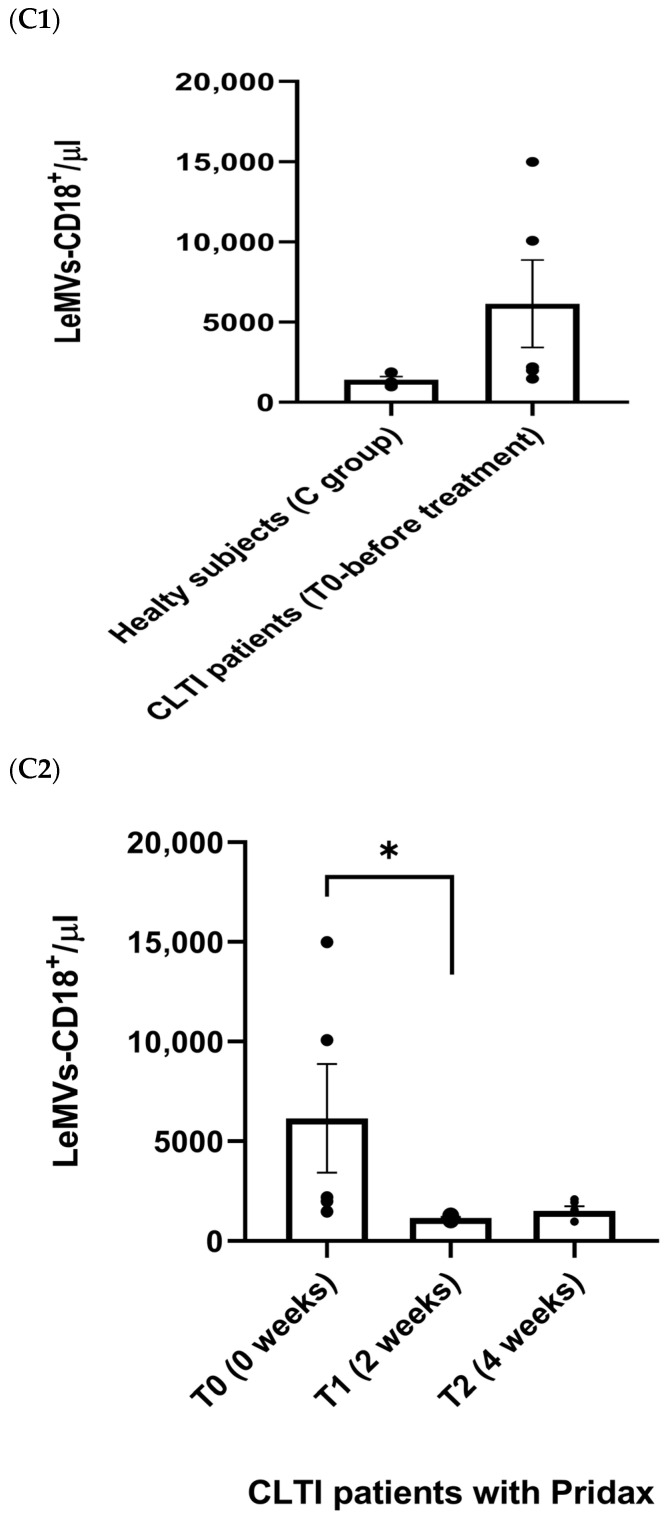

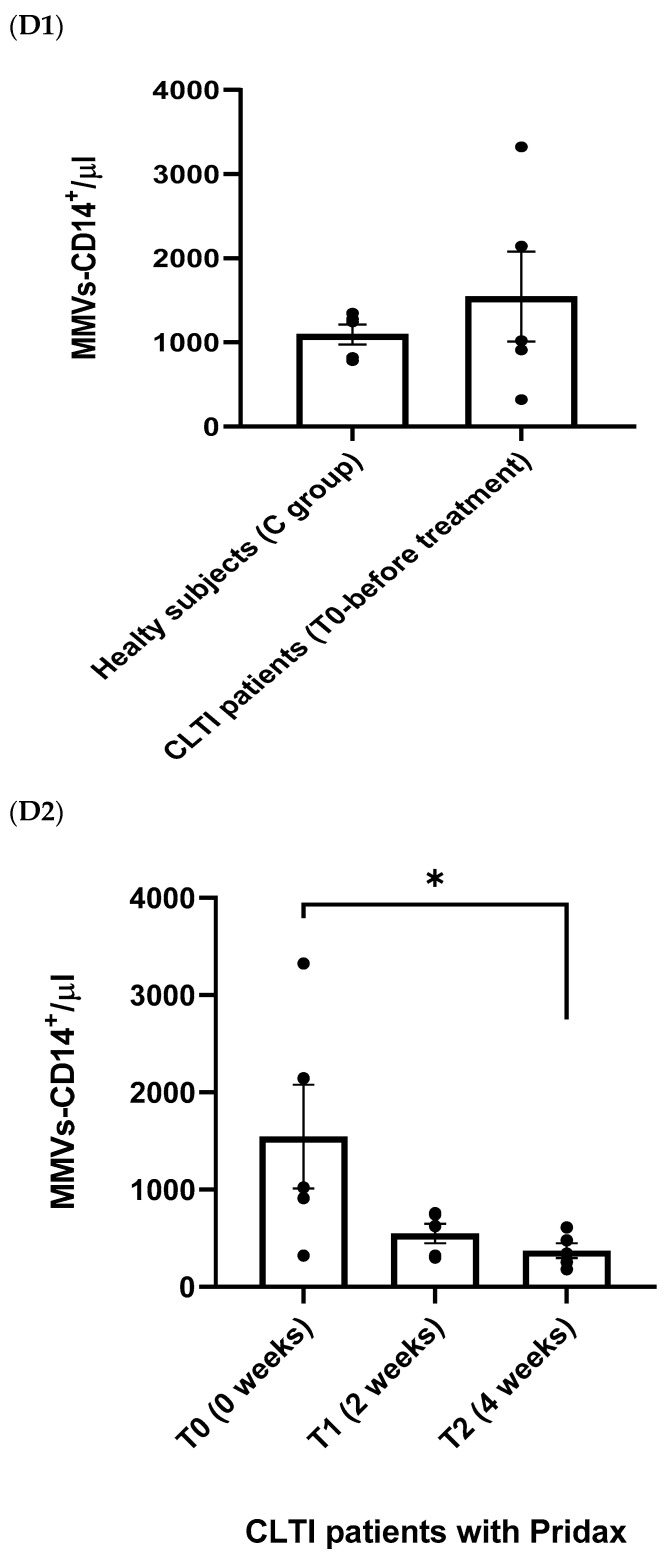

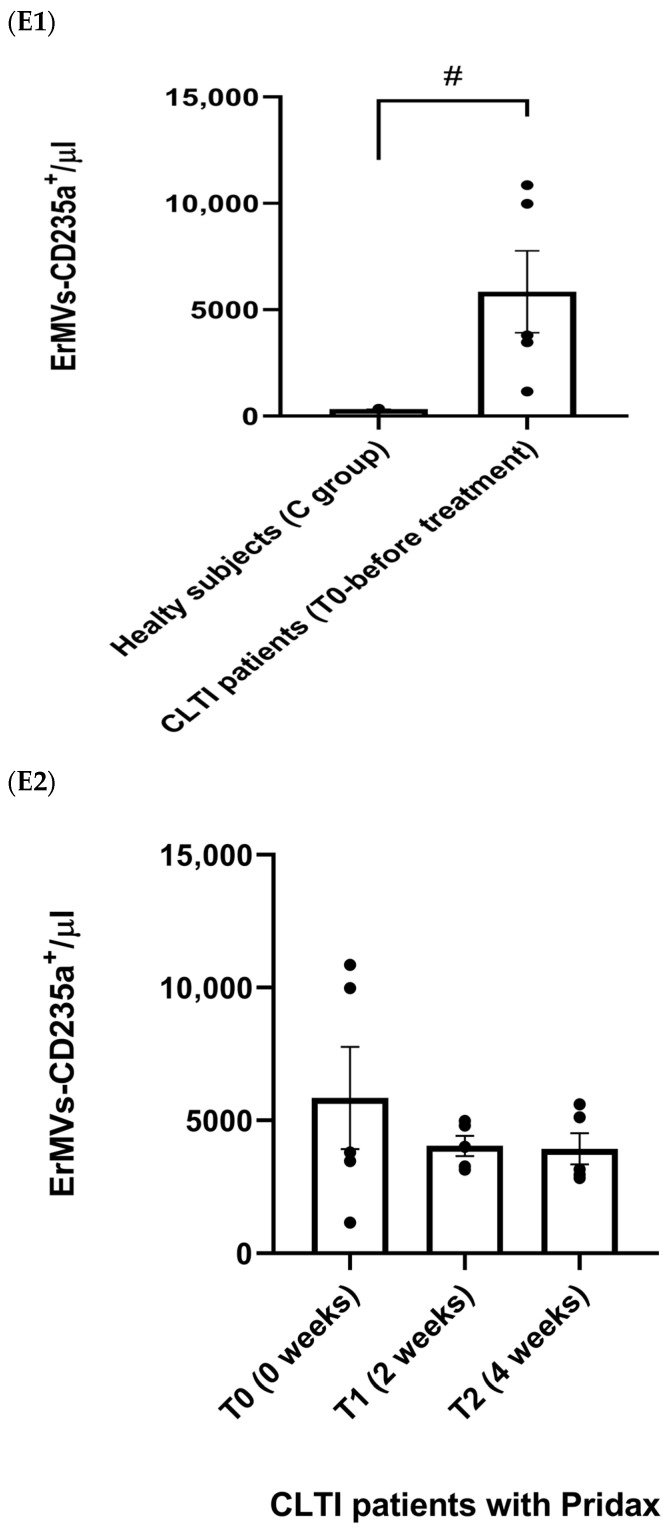
The concentrations of plasma MVs derived from different cell types: MVs originated from platelets, PMVs: AnnexinV^+^/CD41^+^ (**A1**,**A2**); endothelial cells, EMVs: AnnexinV^+^/CD144^+^ (**B1**,**B2**); leucocytes, LeMVs: AnnexinV^+^/CD18^+^ (**C1**,**C2**); monocytes, MMVs: AnnexinV^+^/CD14^+^ (**D1**,**D2**); and erythrocytes, ErMVs: AnnexinV^+^/CD235a**^+^** (**E1**,**E2**), in patients with chronic limb-threatening ischemia (CLTI) treated with alprostadil (Pridax), without revascularization procedures, and healthy subjects. Data are expressed as means ± SEM. * *p* < 0.05, *** *p* < 0.001, (vs. T0), # *p* < 0.05, ### *p* < 0.001 (vs. C group), (one-way ANOVA with Tukey’s multiple comparison test).

The levels of circulating PMVs (CD41+, Annexin V+), EMVs (CD144+, Annexin V+), LeMVs (CD18+, Annexin V+), MMVs (CD14+, Annexin V+), and ErMVs (CD235a+, Annexin V+) were analyzed by flow cytometry in the CLTI patient group treated with alprostadil (Pridax) without revascularization procedures (T0, T1, T2 treatment period), and the control group (Figure 4). The concentrations of plasma-specific MVs were calculated as described in the Section 2.

As shown in Figure 4, CLTI increases significantly circulating levels of EMVs, PMVs, and ErMVs compared to those from healthy individuals’ plasma (Figure 4(A1,B1,E1)).

Also, patients with CLTI who were treated with alprostadil (Pridax) without revascularization procedures exhibited significantly lower levels of plasma PMVs and EMVs after two and four weeks of treatment (T1 and T2 treatment periods), respectively, in comparison to the levels prior to the initiation of treatment (T0 treatment period) (Figure 4(A2,B2)). With respect to the levels of circulating PMVs and EMVs, alprostadil demonstrated its maximal effect following a two-week treatment period (T1), exhibiting a comparable effect to the levels of PMVs and EMVs observed in the plasma of healthy individuals (C group). A significant decrease in LeMV levels was observed following two weeks of treatment (T1 treatment period), while MMV levels exhibited a decrease only at the end of treatment, after four weeks of alprostadil administration (T2 treatment period), in comparison with the initial LeMV and MMV concentrations observed in CLTI patients not receiving alprostadil treatment (T0 treatment period) (Figure 4(C2,D2)).

*This finding suggests that CLTI may be associated with increased circulating levels of EMVs, PMVs, LeMVs, MMVs, and ErMVs compared with healthy individuals. Moreover, alprostadil treatment appears to be associated with a reduction in plasma levels of EMVs, PMVs, LeMVs, and MMVs.*

The evaluation of the temporal changes in these MV subpopulations provides insight into the evolution of inflammatory status, cellular activation, and endothelial dysfunction throughout the treatment period. Circulating MVs are recognized as important biomarkers of vascular injury and thromboinflammatory processes involved in the pathophysiology of CLTI, and their dynamics may reflect the biological response to alprostadil therapy. The comparison of values recorded at T0, T1, and T2 may therefore offer relevant information regarding the effects of treatment on platelet, endothelial, leukocyte, monocyte, and erythrocyte activation, potentially indicating changes in ischemic severity and vascular status over the course of therapy.

**Figure 5:**
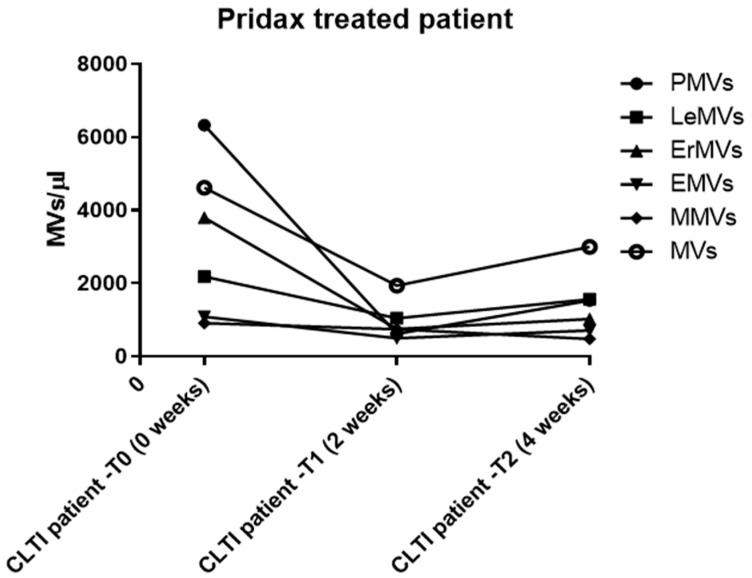
The concentrations of plasma MVs, PMVs, EMVs, LeMVs, MMVs, and ErMVs for a patient with chronic limb-threatening ischemia (CLTI) treated with alprostadil (Pridax), without revascularization procedures, at three distinct moments during treatment: T0 (week 0, prior to treatment administration); T1 (week 2, after two weeks of treatment administration); and T2 (week 4, after four weeks of treatment administration).

Figure 5 illustrates the variation in plasma concentrations of total MVs and their major subtypes—PMVs, EMVs, LeMVs, MMVs and ErMVs—in a patient diagnosed with CLTI who was treated exclusively with alprostadil (Pridax), without undergoing any revascularization procedures. Measurements were performed at three distinct time points during the therapeutic protocol: T0, prior to treatment initiation; T1, after two weeks of treatment administration; and T2, after four weeks of therapy.

Flow cytometry analysis showed that treatment with alprostadil for two and four weeks (T1 and T2 treatment periods), in the absence of revascularization, led to decreased plasma levels of MVs, PMVs, EMVs, LeMVs, MMVs, and ErMVs in a patient with CLTI compared with levels recorded at T0 prior to treatment initiation (Figure 5).

*These results appear to be consistent with the clinical improvements observed in patients with CLTI treated with alprostadil, including enhanced ulcer healing, reduced pain, and improved limb salvage rates.*

*Overall, the findings of the present preliminary study suggest that blood-circulating MVs may have potential as a useful tool for evaluating the efficacy of alprostadil treatment in this patient population.*

## 4. Discussion

The management of patients with advanced peripheral artery disease (PAD), particularly those with chronic limb-threatening ischemia (CLTI), remains a significant clinical challenge. Although revascularization procedures and vasodilator therapies constitute important components of current management strategies, they are not always feasible or sufficient to achieve optimal symptom control and prevent disease progression. In this context, intravenous alprostadil is frequently used as an adjunctive treatment because of its vasodilatory, anti-inflammatory, and antiplatelet properties. However, evidence regarding its clinical benefits in patients with CLTI remains limited and somewhat inconsistent across studies. Therefore, further investigation of the biological effects associated with alprostadil treatment may contribute to a better understanding of its potential role in this patient population.

The results of our preliminary study are in line with clinical observations suggesting that treatment with alprostadil may have potential beneficial effects in patients with CLTI, including pain relief, ulcer healing, and improved limb salvage rates. In this context, the identification of biomarkers that may reflect the biological response to treatment could be of particular importance for monitoring disease progression and assessing therapeutic efficacy.

Circulating microvesicles (MVs) are increasingly recognized as participants in inflammatory and thrombotic processes and as markers of endothelial dysfunction associated with PAD. Assessing MV levels before and during alprostadil treatment may provide insights into the biological processes underlying CLTI and their evolution over time. Additionally, evaluating them in CLTI patients, both with and without revascularization procedures, provides an opportunity to explore potential links between biological changes and clinical progression. In this context, the present analyses were intended to generate hypotheses regarding the potential relevance of MVs in monitoring disease activity and treatment-related biological responses, rather than to establish their clinical utility as biomarkers of therapeutic response.

*In the present preliminary study, patients with CLTI exhibited higher circulating levels of total MVs, as well as several MV subtypes, including endothelial cell-derived microvesicles (EMVs), platelet-derived microvesicles (PMVs), leukocyte-derived microvesicles (LeMVs), monocyte-derived microvesicles (MMVs), and erythrocyte-derived microvesicles (ErMVs), compared with healthy individuals.* These observations are consistent with the hypothesis that MVs may reflect cellular activation and biological processes involved in advanced PAD, including endothelial dysfunction, systemic inflammation, and thrombotic activation. However, given the exploratory nature of the study, these associations should be interpreted cautiously and confirmed in larger studies.

MVs have been associated with inflammation, thrombophilic states, and various cardiovascular complications in patients with coronary artery disease [21,22,23,24,25]. In PAD, MVs have been proposed as potential markers of platelet activation [12]. Among circulating MV populations, PMVs are generally considered the most abundant subtype in patients with PAD [26], and increased PMV levels have been associated with both disease presence and adverse prognosis [13,14]. Furthermore, correlations between circulating PMV levels and plasma coagulation parameters have led to the suggestion that PMVs may help identify PAD patients at increased thrombotic risk [26]. However, the interpretation of PMV measurements requires caution, as previous studies have shown that only PMVs co-expressing P-selectin and CD63 accurately reflect the degree of platelet activation in vitro [14].

Consistent with previous reports [14,16,17,26], we found that EMVs, PMVs, and ErMVs represented the predominant MV subpopulations in the plasma of patients with CLTI. Similar alterations in circulating MV levels have also been described in other pathological conditions, including hypercholesterolemia [11], diabetes [27,28], diabetic dyslipidemia [6], atherosclerosis [3], and cancer [11]. Taken together, these observations are consistent with the notion that circulating MVs may reflect underlying pathological processes across a range of vascular and metabolic disorders. However, further studies are required to determine their specificity and clinical utility as disease biomarkers in PAD.

Additionally, elevated levels of endothelial cell-derived monomeric C-reactive protein (mCRP)-positive MVs have been reported in patients with PAD, while reductions in mCRP-MV levels have been observed following statin therapy [16].

Previous studies have also reported changes in the total number of circulating MVs and in specific MV subpopulations following pharmacological interventions in patients with PAD [29]. For example, treatment with cilostazol has been associated with lower PMV levels [30], whereas atorvastatin therapy has been associated with alterations in PMV subpopulations, including reductions in PMVs expressing P-selectin, tissue factor, and glycoprotein IIIa [31]. Collectively, these observations suggest that circulating MV profiles may be responsive to pharmacological interventions, although the biological and clinical significance of these changes remains to be fully established.

*Furthermore, the present preliminary study found that treatment with alprostadil was associated with lower plasma levels of total MVs and several MV subtypes, including EMVs, PMVs, LeMVs, and MMVs. The most pronounced changes were observed within the first two weeks of treatment.* Specifically, the levels of PMVs in circulation demonstrated a substantial reduction in patients with CLTI following alprostadil administration, with the maximum effect observed after two weeks of treatment. These values were reported in comparison to those observed in healthy subjects. A comparable effect of alprostadil in patients with CLTI was also observed for plasma EMVs. Furthermore, the results of this study indicate that alprostadil administration led to a decline in LeMVs after only two weeks of treatment, while MMV levels exhibited a significant reduction only at the end of the treatment period. Interestingly, previous investigations have reported differing effects of other pharmacological interventions on MV subpopulations, including increased EMV levels following atorvastatin treatment [32], highlighting the complexity of MV biology and the need for additional research in this area.

These observations are consistent with the known vasodilatory and anti-inflammatory properties of alprostadil and may reflect biological processes related to endothelial activation and inflammation in patients with CLTI. The observed decrease in circulating MV levels during treatment appeared to be consistent with the reported clinical improvements in patients, including pain relief and favorable progression of ischemic lesions.

*Collectively, these observations may support a possible association between changes in circulating MV levels and the clinical response to alprostadil therapy.*

However, caution is warranted when interpreting these findings. Given the exploratory nature of the study and the absence of a design specifically intended to establish causal relationships, the observed reductions in circulating MV levels should not be considered direct evidence of a therapeutic effect. Rather, these findings indicate potential biological associations that merit further investigation in larger prospective studies.

On the contrary, the results of the present study indicate that revascularization procedures do not provide an additional benefit in reducing circulating MV levels compared to alprostadil treatment. This observation may indicate that although revascularization restores blood flow at the macrovascular level, inflammatory processes and endothelial dysfunction at the microvascular level may persist or be influenced by systemic factors independent of vascular intervention. Given the exploratory nature of these analyses, these interpretations are speculative and should be considered hypothesis-generating. Further studies are needed to clarify the relationship between revascularization and MV dynamics in patients with CLTI.

*The collective findings suggest that blood-circulating MVs may have potential as biomarkers of disease activity in CLTI and for evaluating the response to alprostadil treatment in patients with CLTI.*

*The continuous observation of MV levels has the potential to offer further insights into the biological mechanisms underlying disease progression and therapeutic response. Consequently, this finding has the potential to further refine therapeutic strategies for this patient population.*

**Study limitations**: In this preliminary study, we evaluated a relatively small number of patients. Since large-population studies have already been conducted, we decided to focus on a more specific, heterogeneous clinical population with a wider range of etiologies. Therefore, this preliminary study concentrated on patients with chronic limb-threatening ischemia (CLTI). The main challenge was distinguishing the combined effects of antibiotics, antiplatelet drugs, anti-inflammatory drugs, statins, and anticoagulants from the effect of alprostadil alone. Patients with PAD often have multiple comorbidities, such as dyslipidemia, diabetes, or hypertension, which may contribute to variability in treatment response.

Additionally, potential beneficial effects of alprostadil may be related to concomitant therapies, including non-prostanoid vasodilators, such as pentoxifylline or cilostazol, administered between alprostadil courses. Lifestyle modifications and risk factor management, such as smoking cessation and improved comorbidity control (e.g., diabetes and dyslipidemia), may have influenced the observed outcomes and cannot be fully separated from the effects of alprostadil.

In the negative control group, which consisted of patients with CLTI who did not receive alprostadil treatment or revascularization, trophic lesions did not progress toward healing during the observation period. Wound management protocols were applied consistently across the study population to ensure reproducibility.

Furthermore, despite the implementation of a random allocation of participants, the present study was designed as an exploratory investigation. Consequently, the findings should be regarded as preliminary and hypothesis-generating rather than confirmatory. Overall, these aspects highlight important limitations of the study design, including the small sample size, lack of a strictly matched control group, potential confounding factors due to concomitant therapies, disease etiology heterogeneity, incomplete quantitative outcome assessment, and limited follow-up. These factors should be considered when interpreting the findings, which support the exploratory nature of this investigation.

In summary, the role of MVs as indicators of treatment response remains to be fully elucidated. Subsequent studies encompassing larger patient populations, lengthier follow-up periods, and meticulously controlled designs are imperative to substantiate these preliminary observations and elucidate whether MV profiling holds clinical relevance in monitoring therapeutic interventions in patients with advanced peripheral arterial disease (PAD).

## 5. Conclusions

The findings of this study suggest that the intravenous administration of alprostadil, according to the applied protocol, appears to be safe for patients diagnosed with CLTI. Furthermore, circulating MV levels may be associated with clinical outcomes. Patients who demonstrated a favorable clinical response tended to exhibit reduced circulating MV levels after one month of alprostadil treatment, accompanied by symptom improvement, including the resolution of rest pain and a lower incidence of major amputations.

However, further investigations involving larger patient cohorts are needed to better define a standardized protocol for the potential clinical use of this proposed biomarker. The execution of such studies could facilitate a more precise characterization of vascular disease severity and potentially enhance the prediction of clinical outcomes following treatment, particularly with regard to the response to alprostadil therapy.

Overall, the assessment of circulating plasma MV levels, including specific subtypes such as EMVs, PMVs, LeMVs, and MMVs, may represent potential biomarkers with possible applicability in vascular surgery practice.

## Figures and Tables

**Table 1:** The clinical characteristics and medical histories of the CLTI patients included in the study. Observation: After finishing alprostadil (Pridax) administration, all the patients were given a prescription for Pentoxifilin Retard (400 mg: 1-0-1).

Patients	Gender/Age (Years)	DM	History of Smoking	HTN	DLD	CAD/CAS	Prior Amputation	Prior Revascularization	Lesion Location	Rutherford/Fontaine Class	Revascularization ± Amputation	Amputation After Pridax	Medication
P1	M/49	No	Smoker	Yes	Yes	Trombangeitis Obliterans	Toe amputation—IV, left hand (previously)	PTA (percutaneous transluminal angioplasty) + left stent in popliteal artery (previously)	Trophic digital ulcers (fingers III, V).	Infrainguinal occlusive diseases. Chronical lower limb threatening ischemia (Fontaine stage IV)	No	Transmetatarsal amputation of right foot (3 months after Pridax)	PLAVIX 75 MG 0-1-0 PENTOXIFILIN R 400 MG 1-0-1 ASPIRIN CARDIO 100 MG 0-1-0 NOLIPREL 5/1.25 MG 1-0-0 CRESTOR 10 MG 0-0-1
P2	M/55	Yes	No	No	Yes	No	Right leg amputation (previously in 2018).	PTA+ left stent “below the knee” (3 years ago)	No	Infrainguinal occlusive diseases. Left pelvic critical limb ischemia (Fontaine stage IV)	No	No	Insulin injection PLAVIX 75 MG 0-1-0 PENTOXIFILIN R 400 MG 1-0-1 ASPENTER 75 MG 0-1-0 VESSEL DUE F 250 ULS 1-0-1 ROSUVASTATIN 10 MG 0-0-1 ESOMEPRAZOL 20 MG 1-0-0 ATACAND 8 MG 0-0-1 METOPROLOL 50 MG 1-0-1/2
P3	F/65	Yes	No	Yes	Yes	Bilateral CAS	No	Personal history of right external iliac artery stent, with a 99% stenosis.	Right calcaneal ulceration	Infrainguinal occlusive diseases. Right lower limb CLTI (Fontaine stage IV)	PTA for stenosis	No	Insulin injection PLAVIX 75 MG 0-1-0 PENTOXIFILIN R 400 MG 1-0-1 ASPIRIN CARDIO 100 MG 0-1-0 VESSEL DUE F 250 ULS 1-0-1 ESOMEPRAZOL 20 MG 1-0-0 SORTIS 40 MG 0-0-1 ATACAND 8 MG 0-0-1
P4	F/79	Yes	No	Yes	Yes	Bilateral CAS	No	Right CEA (carotidian endarterectomy) and patch angioplasty, right ischemic stroke (8 years ago)	Trophic right calcaneal ulceration. Trophic digital ulcers (right foot finger III)	Infrainguinal occlusive diseases. Right lower limb CLTI (Fontaine stage IV)	Above knee amputation	Right calf amputation (4 months after Pridax)	Oral diabetes medication ACENOCUMAROL 2 MG PENTOXIFILIN RETARD 400 MG 1-0-1 CONTROLOC 20 MG 1-0-0 ATACAND 16 MG 1-0-1 DIUREX 50/20 MG DOPEGYT 250 MG 1-0-1 CRESTOR 40 MG 0-0-1 NORVASC 10 MG 1-0-1 NEBILET 5 MG 1-0-0 VESSEL DUE F 250 ULS 1-0-1
P5	F/71	No	No	Yes	Yes	CAD with lesions: PCI (2021), HF NYHA class II, CVI CEAP class C5,HT	No	No	Trophic digital ulcers (right foot finger IV)	Infrainguinal occlusive diseases. Right lower limb CLTI (Fontaine stage IV)	Femoro-popliteal bypass above knee using autologous saphenous vein. Amputation of the right 4th toe	No	ACENOCUMAROL 1MG/2MG ALTERNATIV PLAVIX 75 MG 0-1-0 NEXIUM 20 MG 1-0-0 VESSEL DUE F 250 ULS 1-0-1 PENTOXI R 400 MG 1-0-1 METOPROLOL 25 MG 1-0-0 NOLIPREL 5/1.25 MG 1-0-0 SORTIS 20 MG 0-0-1
P6	M/69	No	Former smoker	Yes	No	CAD with tricoronarian lesions, old MI, stable angina, mild aortic stenosis	No	No	No	Infrainguinal occlusive diseases. Right lower limb CLTI (Fontaine stage IV)	Femoro-femoral bypass with 6 mm collagen impregnated knitted Dacron prosthesis, silver impregnated	No	ACENOCUMAROL 2 MG ASPIRIN CARDIO 100 MG 1-0-0 ENALAPRIL 10 MG 1-0-1 ROSUCARD 5 MG 0-0-1 METOPROLOL 50 MG 1/2-0-1/2 SERLIFT 50 MG 1-0-0 PENTOXIFILIN RETARD 400 MG 1-0-1
P7	M/70		Smoker	Yes	Yes	Peripheral artery disease	No	No	No	Infrainguinal occlusive disease—CLTI (Fontaine stage IV)	Amputation of the left halux	No	PENTOXIFILIN RETARD 400 MG 1-0-1 ASPIRIN CARDIO 100 MG 1-0-0 VESSEL DUE F 250 ULS 1-0-1 SORTIS 20 MG 0-0-1 OMEZ 20 MG 1-0-0
P8	M/60 y	Yes	Smoker	Yes	Yes	No	Yes (halux)	No	Halux and forefoot	Infrainguinal occlusive disease—CLTI (Fontaine stage IV)	Above knee femoro-popliteal bypass. Amputation of the right halux	No	PENTOXIFILIN RETARD 400 MG 1-0-1 ASPIRIN CARDIO 100 MG 1-0-0 VESSEL DUE F 250 ULS 1-0-1 SORTIS 20 MG 0-0-1 OMEZ 20 MG 1-0-0
P9	M/69	Yes	No	Yes	Yes	Yes	Yes (halux)	No	Halux	Infrainguinal occlusive disease—CLTI (Fontaine stage IV)	No	No	PENTOXIFILIN RETARD 400 MG 1-0-1 ASPIRIN CARDIO 100 MG 1-0-0 VESSEL DUE F 250 ULS 1-0-1 SORTIS 20 MG 0-0-1 OMEZ 20 MG 1-0-0
P10	M/65	No	Yes	Yes	Yes	No	No	No	Perimaleolar	Infrainguinal occlusive disease—CLTI (Fontaine stage IV)	PTA + stent iliac artery	No	PENTOXIFILIN RETARD 400 MG 1-0-1 ASPIRIN CARDIO 100 MG 1-0-0 VESSEL DUE F 250 ULS 1-0-1 SORTIS 20 MG 0-0-1 OMEZ 20 MG 1-0-0

Obs. All the patients were given prescribed PENTOXIFILIN R 400 MG 1-0-1 after finishing Pridax administration. DM = diabetes melitus; HTN = hypertension; DLD = dyslipidemia; CAS = carotid artery stenosis; CAD = coronary artery disease; PCI = percutaneous coronary intervention; HF = heart failure; CVI = chronic venous insufficiency; HT = hyperthyroidism; MI = myocardial infarction; PTA = percutaneous transluminal angioplasty.

**Table 2:** The key monitored plasma parameters for CLTI patients before treatment with alprostadil.

Patients	Gender (F/M)	Age (Years)	Mean Platelet Volume (fL)	Platelet Number (Cells/µL)	White Blood Cells (10^9^/L)	Haemoglobin (g/dL)	Blood Glucose (mg/dL)	Cholesterol (mg/dL)	LDL-Cholesterol (mg/dL)
N = 7	M	49–69	10.10 ± 0.9	285.67 ± 48.9	9.46 ± 2.3	13.90 ± 2.0	90.67 ± 21.5	187.00 ± 29.1	94.67 ± 47.6
N = 3	F	65–79	10.53 ± 0.9	457.00 ± 86.2	10.63 ± 2.1	12.60 ± 1.1	121.33 ± 33.9	146.67 ± 41.5	53.00 ± 35.4

## Data Availability

The original contributions presented in this study are included in the article. Further inquiries can be directed to the corresponding author(s).

## Abbreviations

The following abbreviations are used in this manuscript:

CLTI	Chronic Limb Threatening Ischemia
PGE1	E1prostaglandin
MVs	Microvesicles
PAD	Peripheral Artery Disease
ABI	Ankle Brachial Index
PTA	Percutaneous Transluminal Angioplasty
